# A systematic review of the distribution of take-home naloxone in low- and middle-income countries and barriers to the implementation of take-home naloxone programs

**DOI:** 10.1186/s12954-022-00700-x

**Published:** 2022-10-20

**Authors:** Hawraa Sameer Sajwani, Anna V Williams

**Affiliations:** 1 Department of Addictions, Institute of Psychiatry, Psychology and Neuroscience, King’s College London, London, UK; 2 University of Adelaide, Adelaide, Australia; 3 Virginia Commonwealth University, Richmond, VA US; 4 University of California, Los Angeles, Los Angeles, CA US; 5 Sheikh Khalifa Medical City, Abu Dhabi, UAE

**Keywords:** Opioid use, Opioid overdose, Opioid overdose death prevention, Take-home naloxone, Intranasal naloxone, Low- and middle-income countries

## Abstract

**Background:**

Opioid overdose epidemic is hitting record highs worldwide, accounting for 76% of mortality related to substance use. Take-home naloxone (THN) strategies are being implemented in many developed countries that suffer from high opioid overdose death rates. They aim to provide overdose identification and naloxone administration training, along with THN delivery to opioid users and others likely to witness an overdose incident such as family members and peers. However, little is known about such measures in low- and middle-income countries (LMIC), where opioid use and opioid-related deaths are reportedly high. This systematic literature review aims to examine the distribution of THN in LMIC, review studies identifying barriers to the implementation of THN programs worldwide, and assess their applicability to LMIC.

**Methods:**

The literature was searched and analyzed for eligible studies with quality assessment.

**Results:**

Two studies were found from LMIC on THN programs with promising results, and 13 studies were found on the barriers identified in implementing THN programs worldwide. The main barriers to THN strategies were the lack of training of healthcare providers, lack of privileges, time constraints, cost, legislative/policy restrictions, stigma, fear of litigation, and some misperceptions around THN.

**Conclusions:**

The barriers outlined in this paper are probably applicable to LMIC, but more difficult to overcome considering the differences in their response to opioid overdose, their cultural attitudes and norms, the high cost, the waivers required, the legislative differences and the severe penalties for drug-related offenses in some of these countries. The solutions suggested to counter-act these obstacles can also be more difficult to achieve in LMIC. Further research is required in this area with larger sample sizes to provide a better understanding of the obstacles to the implementation, feasibility, accessibility, and utilization of THN programs in LMIC.

## Background

The opioid epidemic is alarming in many countries with record drug-related death rates. Opioid misuse is highly prevalent in North America, Europe, East and South Asia, North Africa and the Middle East [1–3]. Several of the Islamic and Arab world countries and the Middle-Eastern countries have opioids as the primary drug of choice among persons treated for substance use problems with 94.9% of the drug using population in Syria using opioids, 64.3% in the United Arab Emirates, 42.7% in Egypt, 41% in Oman, 38.5% in Lebanon, 31.2% in Kuwait, 8.1% in Saudi Arabia and 3.6% in Jordan [1, 2].

Iran, Pakistan, Iraq, Qatar and the United Arab Emirates were the main countries that saw an increase in heroin use in 2015 and 2016 [1]. This increase was linked to the expanding of heroin trafficking from Afghanistan to these countries. Other countries such as Saudi Arabia, Jordan and Israel saw stabilization in heroin use, as stimulants are playing a larger role in these countries currently. On the other hand, some countries in East and South-East Asia saw a decline in heroin use in 2016 such as China, Indonesia, Thailand, the Republic of Korea and Hong Kong [1].

In one year, from 2016 to 2017, global opium production increased by 65% to 10,500 tons in 2017, which’s the highest estimate recorded by UNODC. Opium yields from Afghanistan only reached 9000 tons, which was mainly the result of a gradual increase in poppy yields [1]. This has led to a huge increase in global opium production, mixed with heroin or other drugs, by 65% from 2016 to 2017 and by 120% since 2015 [1]. In 2016, the global seizure of pharmaceutical opioids such as tramadol, which is a prescription analgesic opioid for moderate to severe pain relief, was 87 tons, around the same as the amount of heroin seized that year [1].

In 2017, the percentage of world total seizures of opium in Asia was 98% from near and Middle East/ South-West Asia with the highest percentage seized from Iran amounting to 80%. This was followed by 10% from Pakistan and thirdly 8% from Afghanistan. On the other hand, the percentage of world total seizures of heroin and morphine was also the highest from near and Middle East/ South-West Asia countries, reaching 76%, with Afghanistan amounting for 33%, Iran 20%, Pakistan 19%, China 6%, then followed by other regions such as the United States 5%, Turkey 4%, Bahrain 3%, and India 1% [1]. As a result, 92.8% of the drug using population in Afghanistan use opioids, 81.2% in Azerbaijan, 50.3% in Kazakhstan, 91.8% in Tajikistan, 95.1% in Turkmenistan, 57.4% in Kyrgyzstan, 51.8% in Uzbekistan, 83.0% in Myanmar, 76.9% in Bangladesh, 95.1% in Armenia, 13.3% in Mongolia, 12.1% in Singapore, 82.1% in Sri Lanka, 37.9% in Malaysia, 58.6% in Turkey, 100% China and Israel, Maldives 87.0% and 49.2% in India [1].

In Africa, opioids are the leading cause of drug-related mortality in a large number of countries such as South Africa, Kenya and the Seychelles. Opioids are also the primary drug among persons treated for substance use problems there, with 100% of the drug using population in United Republic of Tanzania using opioids, 70.6% in Mozambique, 39.4% in Nigeria, 80.6% in Mauritius, 11.5% in Eritrea, 18.8% in Ethiopia, 7.3% in Madagascar, 5.1% in Morocco, 6.5% in Senegal, 45.0% in the Seychelles and 17.3% in South Africa [1]. This is likely reflecting the effect of heroin trafficking from South-West Asia along the southern route. In terms of pharmaceutical synthetic opioids, tramadol is the drug of choice that’s being increasingly misused in West and North Africa and the near and Middle East in Asia. Global seizures of tramadol are now mostly reported from African countries accounting for 87% of the total global seizure of opioids in 2016; overriding countries in Asia, which previously had more than half the global seizures of opioids [1].

The rate of opioid users who overdose over a lifetime reaches up to 70% [4]. History of a previous overdose is a strong predictor of the possibility of subsequent overdoses, and the risk increases with each overdose [5]. Other risk factors for overdose in people using prescription opioids are high prescription doses, male gender, co-prescription with other depressogenic medications (e.g., benzodiazepines), co-occurring psychiatric disorders and lower socioeconomic status [4, 6]. People who reinstate opioid use in the first few weeks following a period of abstinence are at a heightened risk of overdose as a consequence of reduced or lost tolerance to opioids [4, 6, 7]. This can happen after a recent release from incarceration [4, 6], or a recent discharge from a controlled environment such as an inpatient or residential detoxification center [7], or cessation of medication-assisted treatment with opioid antagonists such as naltrexone.

Several mechanisms have been introduced to address the underlying causes of opioid overdose, such as monitoring opioid prescribing practices, restrictions and sanctions on inappropriate opioid prescribing, monitoring and prohibiting inappropriate over-the-counter sales of opioids, and expanding the treatment of opioid dependence to cover illicit and prescription opioid users worldwide [6]. Some of the other measures to combat this problem are needle and syringe exchange programs. Of the 179 countries with evidence of injecting drugs, needle and syringe programs are known to be available in 93 countries of them, which comprises 52% only [1]. Opioid substitution therapy is another important treatment alternative; however, it is available in 86 countries only, which is 48% [1]. Besides, there are 79 countries implementing both needle and syringe programs and opioid substitution therapy, which is 44% only, with only four of them (three in Western Europe and one in Oceania) providing high coverage of both needle and syringe programs and opioid substitution therapy [1].

One of the main effective strategies to reduce the risk of fatal opioid overdose is the wider distribution of take-home naloxone (THN) kits to opioids using patients, their family members and peers [4, 5, 8, 9]. Naloxone has also been included in the WHO Model List of Essential Medicines [9]. Over the past 20 years, THN strategies have been implemented in many countries worldwide, providing naloxone training and overdose management education, as well as THN kits to opioid users and others likely to witness an opioid overdose incident [10]. Almost 3 in 4 of people who use heroin or inject drugs report that they have witnessed an overdose event, whether a fatal or a nonfatal one [1]. This shows that people who use drugs could have a vital role in preventing a fatal overdose.

Opioid overdose incidents usually occur in private homes and are mostly witnessed by a partner, a family member, or close peers [1], which further emphasizes the importance and necessity of THN program implementation worldwide, covering all countries affected by this epidemic. Educating patients, family members, and generally the layperson about the risk of opioid overdose and the use of THN is important to provide them with the knowledge and tools to deal with overdose emergencies confidently [11, 12]. Even if they were to administer naloxone to a non-opioid overdose case, naloxone would not be harmful, as it is known to be safe and will probably only cause a short-lived withdrawal period in case the receiver was an opioid dependent patient. In addition, naloxone should be readily accessible, whether intramuscular or intranasal formulation, for all emergency and medical staff as well as law-enforcement personnel [13].

Several studies have shown that THN programs are associated with decreased fatal overdose events among the at-risk population [14]. A study of 152,283 naloxone kits provided to laypersons from 1996 to mid-2014 in the United States recorded 26,463 opioid overdose reversals over these years [15]. Another study found that 9% of naloxone kits distributed are likely to be used in a peer opioid overdose reversal measure within the first three months of supply [16]. Nevertheless, this does not mean that the other 91% were wasted and could have been kept for future use. A systematic review revealed that one death was reported amongst every 123 opioid overdose patients who were administered with THN [17], while one fatal overdose was reported in every 20 overdose events by UNODC [18]. In a cost-effectiveness study of THN programs, they estimated that a distribution of THN reaching 30% of heroin users can prevent around 6.6% of overdose fatalities [4]. This would amount to the prevention of 2,500 premature deaths in a population of 200,000 heroin users [4].

## Methods

Little is known about harm reduction measures such as THN distribution in the developing world or low- and middle-income countries (LMIC). Identifying barriers to the implementation of THN strategies is essential to come up with an effective and practical change plan, which can be implemented in developing countries. One strategy can be investigating barriers to the implementation of THN programs worldwide, including countries that already have established THN distribution programs, and then assess their applicability to LMIC, estimating the barriers that can be shared between both worlds. The first aim of this study was to systematically review the scientific literature on the distribution of THN in LMIC, and the results found in terms of efficacy, number of THN kits used, and opioid overdose fatalities prevented. The second aim was to investigate the barriers and obstacles identified to the implementation of THN programs worldwide.

Since a low number of studies were expected to be found, all empirical studies, cohort studies, and cross-sectional analyses such as surveys involving the use of THN were included, as well as studies with small samples. Studies looking at other harm reduction strategies such as opioid replacement therapy were excluded. The studied population included all types of opioid users, whether illicit or prescription opioid users. Studies focusing on family members of opioid users, peers, significant others, and laypersons were included as well. Articles were excluded if they just spoke about naloxone in general without a focus on THN.

The search tools that were used were Medline/PubMed, PsycINFO, and Google Scholar. The gray literature was explored as well. Virginia Commonwealth University library search tool was used to obtain full text articles. The main keywords used were (Naloxone), (take-home naloxone), (naloxone kits), (low-income countries), (middle-income countries), (poor countries), (developed countries), (developing countries), (Naloxone barriers) and (opioid overdose prevention) in various combinations. Some of the countries known to have an opioid endemic with low- or middle-income were searched separately with (Naloxone) as well. The following MeSH terms were utilized: Drug Overdose/drug therapy, Drug Overdose/epidemiology, Drug Prescriptions, Drug Utilization/trends, Narcotic Antagonists/therapeutic use, Naloxone/administration and dosage, Naloxone/therapeutic use, Narcotic Antagonists/administration and dosage, Narcotic Antagonists and Narcotics/poisoning.

**Fig. 1:**
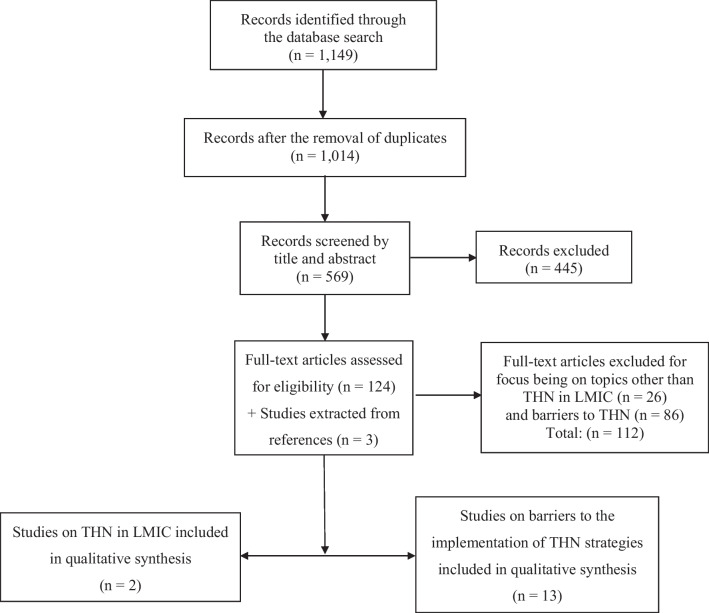
Adapted PRISMA Flow Diagram

The PRISMA chart [19] shown below was used to demonstrate a simplified chart of the process of paper selection, inclusion and exclusion (Fig. 1). Some of the articles covering the barriers to THN strategy implementation discussed other aspects of opioid overdose prevention, so they were partly eligible and included. All papers included were screened for the presence of any conflict of interest and for relevant IRB approvals [20]. The overall quality of each of the included studies was appraised using an adapted CASP checklist [20]. All studies included in this paper passed the CASP checklist.

## Results

### Take-home naloxone in low- and middle-income countries

Only two studies were found from LMIC on THN programs with the results of their implementation. None were found from Middle-Eastern countries. This in itself is a finding that points out to the lack of such interventions such as THN in these countries, and hence the paucity of research on it.

The first paper was a cohort study in Kyrgyzstan and Tajikistan focusing on the number of kits reported to be received by opioid users or administered to others by them and the number of wasted distributed naloxone ampoules. This was calculated through brief surveys upon returning of people who inject drugs for more ampoules [21]. And the second one was a randomized controlled trial based in Kazakhstan held in between 2009 and 2013, and reported the use of naloxone in opioid overdose prevention and HIV/HCV prevention programs [22] However, they focused on other opioid use-related outcomes, and just reported the number of participants who used naloxone to reverse an opioid overdose or had someone else administer it to them [22].

The first study was published in 2011 and it looked at the usage and wastage of distributed naloxone kits [21]. The participants were 158 from Kyrgyzstan and 59 from Tajikistan. The participant reports showed that of the ampoules received in both pilot projects, around 46% in Kyrgyzstan and 78% in Tajikistan were used, and only 3% of these ampoules were wasted in both countries [21]. The low wastage rates show the high rate of utilization of the distributed naloxone supply either by consumption or by being kept for future use, since a portion of the participants from Kyrgyzstan reported keeping the ampoules for future use in addition to using some of them [21].

The second study was an RCT conducted between 2009 and 2013 on 479 opioid users [22]. It was an evaluation study of the efficacy of a couple-based integrated Skills and Knowledge on Overdose Prevention (SKOOP) program and HIV/HCV prevention intervention versus a general wellness promotion and overdose prevention program [22]. Naloxone administration training and distribution of THN was provided to both arms of the study through 5-session, and the results showed that 105 participants used naloxone to reverse an opioid overdose or had someone else administer naloxone to them during the study period in both arms collectively [22]. All these cases were saved, except one case that was intoxicated by alcohol as well and eventually died. They also have a common but ineffective practice of injecting saline to reverse overdose in this region, which was noticed to be significantly reduced in both arms [22].

### Barriers to the implementation of THN strategies

A total of 13 papers were found on the barriers and obstacles faced in implementing THN programs worldwide. The studies were conducted between 2005 and 2018 and were mostly from the US and the UK (Table 1). The studies tested mainly knowledge, opinions, attitudes, and perceptions of healthcare providers and opioid users on overdose education and THN delivery. They also addressed higher administrative issues such as the policy and regulations governing THN delivery. The cohort studies compared attitudes and utilization, pre-and post-education and training on THN delivery. A list of the 13 papers included in this systematic review and their methodologies are displayed in (Table 1). After thoroughly assessing the papers that were included in this review, the main barriers identified to the implementation of THN strategies were compiled and constructed in the following main themes that are presented in (Table 2).

**Table 1:** Details of the 13 papers included in this systematic review of barriers to the implementation of THN strategies

Author	Study Title	Year	Study Methodology	Region
Barbour et al. [40]	Emergency physician resistance to a take-home naloxone program led by community harm reductionists	2018	Cohort study	California, Irvine, US
Beletsky et al. [32]	Physicians’ Knowledge of and Willingness to Prescribe Naloxone to Reverse Accidental Opiate Overdose: Challenges and Opportunities	2007	Cross-sectional survey	United States
Carpenter et al. [36]	Factors Associated with How Often Community Pharmacists Offer and Dispense Naloxone	2018	Cross-sectional survey	North Carolina, US
Davis et al. [43]	Legal Changes to Increase Access to Naloxone for Opioid Overdose Reversal in the United States	2015	Review article	United States
Drainoni et al. [46]	Why is it so hard to implement change? A qualitative examination of barriers and facilitators to distribution of naloxone for overdose prevention in a safety net environment	2016	Qualitative study	Boston, US
Gaston et al. [60]	Can we prevent drug related deaths by training opioid users to recognise and manage overdoses?	2009	Cohort study	Birmingham and London, England
Hammett et al. [38]	Pharmacies as providers of expanded health services for people who inject drugs: A review of laws, policies, and barriers in six countries	2014	Qualitative study	U.S., Russia, Vietnam, China, Canada and Mexico
Khatiwoda et al. [64]	Facilitators and Barriers to Naloxone Kit Use Among Opioid-Dependent Patients Seeking Treatment at Medication Assisted Therapy Clinics in North Carolina	2016	Cross-sectional survey	North Carolina, US
Pricolo et al. [41]	Naloxone Rescheduling in Australia: Processes, Implementation and Challenges with Supply of Naloxone as a “pharmacist Only” over-the-Counter Medicine	2018	Review article	Australia
Sondhi et al. [42]	Stakeholder perceptions and operational barriers in the training and distribution of take-home naloxone within prisons in England	2016	Qualitative study	England
Tobin et al. [30]	Attitudes of emergency medical service providers toward naloxone distribution programs	2005	Cross-sectional survey	Baltimore, US
Tobin et al. [65]	Awareness and Access to Naloxone Necessary but Not Sufficient: Examining Gaps in the Naloxone Cascade	2018	Cross-sectional survey	Baltimore, US
Winograd et al. [31]	Medical providers' knowledge and concerns about opioid overdose education and take-home naloxone rescue kits within Veterans Affairs health care medical treatment settings	2017	Cross-sectional survey	United States

**Table 2:** Barriers to the implementation of THN strategies and the articles reporting them

Barriers to the implementation of THN strategies	Studies reported
Lack of physician knowledge and willingness	Beletsky et al. [32] Tobin et al. [30] Winograd et al. [31]
Physician time constraints and inadequate staffing	Barbour et al. [40]
Pharmacist time constraints, misconceptions, and lack of training	Barbour et al. [40] Carpenter et al. [36] Hammett et al. [38]
Inadequate policy development	Davis et al. [43] Drainoni et al. [46] Sondhi et al. [42]
Prescription policy restrictions	Davis et al. [43] Pricolo et al. [41]
Perceived lack of comprehension or misinterpretation by opioid users	Beletsky et al. [32] Carpenter et al. [36] Sondhi et al. [42] Tobin et al. [30]
Stigma and fear of litigation	Davis et al. [43] Gaston et al. [60] Sondhi et al. [42]
Difficulties perceived by opioid users in carrying the naloxone kit	Gaston et al. [60] Khatiwoda et al. [64] Tobin et al. [65]

## Discussion

### THN in low- and middle-income countries

Very few studies were found on THN programs in LMIC. This could be attributed to the small number of THN programs in LMIC, and them being fully dependent on emergency medical services to use naloxone when required. However, the two studies found have shown remarkably good results from implementing THN strategies [21, 22], supporting expanding this practice to at-risk populations in developing countries. The very low naloxone supply wastage and the high consumption reported by Kan and colleagues [21] suggests that the demand for naloxone is high, and highlights the opioid use and overdose problem in such countries. Gilbert and colleagues’ results also showed that a high number of participants used naloxone to reverse an overdose incident successfully, or had someone else administer naloxone to them during the study period [22]. Their findings highlight the opioid use and overdose problem, and support the safety and feasibility of implementing THN programs in LMIC [22]. However, considering the limited number of studies found from LMIC, it is questionable whether any generalization to LIMIC can be inferred, and the applicability of the results is further explored in the end of this review.

#### The burden of overdose in LMIC and the lack of naloxone supply

There have been some efforts to identify the main barriers that hinder efforts to make THN available in LMIC. Studies about naloxone in Kazakhstan, Kyrgyzstan and Uzbekistan reported that the lack of supply of naloxone in these countries was due to its limited shelf life, low pharmaceutical profits, failure to register naloxone in governmental purchase lists, and the lack of training of medical professionals on naloxone use in these countries [23, 24]. In another study from Malaysia of a cohort of 460 people who inject opioids, it was found that 43.3% had ever overdosed [25]. HIV and opioid overdose problems occurring simultaneously in LMIC also highlight the importance of expanding combined naloxone distribution and HIV prevention programs in these countries for better health outcomes [26–29].

### Barriers to the Implementation of THN Strategies

#### Physician Education and Training

Most of the studies included in this analysis emphasize the importance of harm reduction education to healthcare providers [30, 31]. They recommend physician training by professional organizations or during medical school and residency training [32, 33]. They also emphasize the importance of alleviating medicolegal anxiety about prescribing THN [32]. Some surveys reported that healthcare providers are showing poor attitudes and low willingness to participate in THN distribution [30, 34]. Attitude, confidence, training, self-efficacy, knowledge, time, and institutional support all play key roles in encouraging the use of THN [35], and even a few committed physicians can make a big difference [35].

#### Pharmacist training and prescribing privileges

Pharmacists play a vital role in the implementation of THN strategies. Barriers that were identified to pharmacists providing naloxone education included time constraints, inadequate training, perceived lack of patient comprehension [36, 37], and lack of pharmacist prescribing privileges [38, 39]. A collaborative practice approach between prescribing physicians and pharmacists can be adopted to solve this issue by providing pharmacists with the necessary privileges to dispense naloxone through pre-authorized orders on the electronic health record system that they may activate. Stakeholders can play an important part in facilitating such policy reform efforts, and fighting community stigma and resistance [38, 39]. Targeted naloxone training in smaller chain pharmacies may be necessary as well [40], and structural changes in the level of insurance coverage to compensate pharmacists for brief interventions such as counseling on substance use, and naloxone administration training may be required as well [38].

#### Policy and regulation development

In addition to granting pharmacists prescribing privileges, THN dispensation should be permitted in community pharmacies as well allowing community pharmacists to prescribe as well, and not only hospital or clinical pharmacists [41]. This can help significantly remove accessibility barriers to THN distribution [39]. There is also a need to work on prison policies to commence THN delivery education there, and senior prison staff can be assigned this responsibility [42]. Statutory regulations should be clearly delineated, specifying the group of individuals that can receive a prescription of naloxone, and whether nonmedical staff can dispense the medication or not. They should also provide immunity to those who prescribe, dispense, and administer naloxone, or report a suspected overdose emergency [31, 43–45]. Additionally, the option to prescribe naloxone and allow patients to pick it up at an outpatient pharmacy, or on discharge from an inpatient facility, or have it available on-site at the ED to distribute to patients could all be effective methods to increase the chances that a patient will walk away from the hospital with the kit in hand [40, 46].

#### Cost-effectiveness of THN programs

A major obstacle postulated in implementing THN programs in LMIC was the perception of it being costly [28]. Studies in the US [5, 47] and the UK [4] have found the distribution of THN to be cost-effective and much cheaper than the large costs incurred from mortality rates associated with opioid overdose [5, 29]. Many legislations in various cities in the US have included naloxone in their basic life support measures [47]. Some countries have free supply at the point of distribution such as in the UK where no cost is incurred on the recipient, as it is paid through taxes and the consequent funding of public services.

However, worth considering is whether this could be generalizable internationally, especially to LMIC, since the mechanisms of how health costs are being covered may differ. Many of these countries might not be able to afford naloxone to be stocked in hospitals, ambulances or to make it available to the community in the form of THN kits. This is mainly considering the possibility that a good amount of that supply may be wasted, since the studies that were found in this paper from LMIC cannot be considered as representative of all LMIC [21, 22]. Hence, measures for the cost-saving utility of THN in LMIC need to be further explored.

#### Overdose education and THN training to opioid users

In November 2014, new WHO guidelines stated that naloxone should be made available to anyone at risk of witnessing an overdose [48, 49]. However, it is being feared that the distribution of THN to at-risk populations might condone or promote opioid misuse [42], and may reduce the perceived negative consequences associated with opioid and polysubstance use, leading to riskier patterns of use [31, 50]. There were also concerns about administration mistakes and appropriate disposal of ampoules. This perceived lack of comprehension by opioid users could also read more like provider stigma and discrimination, where opioid users are viewed as lacking the required skill and comprehension to take such a responsibility [30].

Although studies are reassuring that opioid users can be trained to respond appropriately to opioid overdose incidents and save lives, there are many cognitive, practical, and emotional factors that influence one’s response in such situations [51], such as their previous experiences with overdose [49]. Nevertheless, there’s no strong evidence suggesting increased drug use following THN/overdose training among heroin users [54]. Contrary to that, following training, good competencies were demonstrated in identifying signs and symptoms of overdose, calling for help, applying basic resuscitation and first aid techniques, administering naloxone, and providing post-resuscitation support [49, 52, 53]. Since naloxone has a short half-life and its effect can wear off quickly, therefore, providers should be instructed on how to repeat administration when necessary to prolong the antagonizing effect to the comparatively longer half-life of the intoxicating opioid. [48].

#### Overdose education and THN training to family members and the layperson

Provision of education and training to opioid users, peers and family members are essential tools in the expansion of THN initiatives to combat the risk of overdose deaths [53, 55, 56]. A survey that looked at the charts of 312 patients in the US that received naloxone found that 213 of them had their first dose administered by a family member, a bystander, police or by BLS personnel. Small pilot peer-administered naloxone overdose prevention projects in LMIC countries such as Kazakhstan, Kyrgyzstan and Uzbekistan are being conducted as well [29].

#### Overdose education and THN training to at-risk special populations

The duration of imprisonment is a unique opportunity to educate inmates about overdose response and THN delivery [42]. People who inject drugs with a history of incarceration are more likely to encounter an opioid overdose than those never incarcerated [42, 57]. They are also more willing to utilize naloxone in case of an opioid overdose event, so providing training along with THN to discharged inmates can be a crucial intervention [42, 57]. A good option can also be to deliver training sessions on an outreach basis through satellite locations to various targeted high-risk groups, such as youth or juvenile centers, recovery groups, offender services, prisons and community centers [57].

THN training can be provided to homeless people as well, as they can comprise a big portion of the opioid using population and their peers. Studies have shown that several of them are able to recognize signs of heroin overdose and are willing to take the initiative in a rescue measure such as administering naloxone, provided they have received prior training and education [58–60]. Another special population that can be reached by naloxone distribution programs are opioid using pregnant women that are at risk of an overdose. However, special precautions should be followed, as this is a complex population and WHO has issued guidelines on managing opioid overdose in pregnancy with naloxone. They recommend to start with the lowest possible dose (400 μg) to reduce the risk of acute withdrawal that could induce seizures and possibly death in the fetus [61].

#### Alleviation of concerns of legal responsibility

Many individuals that received naloxone stock kept it at home and did not have it available at the time of an overdose [60]. They were essentially concerned about police involvement at the scene of an overdose, the stigma of carrying injectable material if searched, and fear of being treated as responsible when naloxone is used [60]. These concerns are shared by prisoners as well that were worried that carrying naloxone would be perceived as a sign of lack of commitment to recovery [42]. These concerns are valid as many emotional and social factors influence one’s response in such situations of an overdose event, such as their confidence, willingness, compassion to help others, and their ability to make decisions and communicate effectively [51]. Integrating education about prescription laws during THN training can help alleviate these concerns of being searched by police when emergency services are contacted, while they’re in possession of naloxone [60].

#### Seeking emergency help during an overdose event

It is important to encourage overdose witnesses to seek help from emergency responders in the event of an overdose [43]. Unfortunately, witnesses of heroin overdose often do not call 911 [32], and generally, the practice of seeking emergency medical help in the event of an overdose is not always followed [62, 63]. This further emphasizes the importance of allying concerns of legal liability, and authorities ought to consider legal reforms that provide immunity from drug possession or drug use charges for overdose responders [60].

#### Difficulties perceived by opioid users in carrying the naloxone kit

Naloxone kits are perceived by some consumers to be too large, bulky, and uncomfortable to be fitted in a pocket [60, 64]. This can be a deterrent from carrying it for the possibility of encountering an overdose [65]. The use of various naloxone formulations and packaging methods such as autoinjector intramuscular delivery systems can be easier to administer for nonmedical users and can be manufactured in a more compact way to fit pockets easily.

### Translation and applicability of the results to LMIC

In the end of this discussion, it appears that most of the barriers identified in this review, the solutions and remediation strategies suggested could possibly be applicable, appropriate, and feasible for LMIC. However, most of the obstacles to THN strategies that are identified in this paper are found in high-income countries mainly the US and UK, so this raises several questions in terms of their applicability and generalizability to LMIC. This is mainly considering how LMIC may differ in their response to opioid overdose and their cultural attitudes and norms. Some LMIC may have different regulatory frameworks and legislative systems which could make the availability of medicines such as THN without physician prescription more difficult, especially if they do not have the concept of community pharmacies. Furthermore, the stance of physicians on prescribing THN may differ also, considering the stigma and fears of misinterpretation of such a strategy as condoning opioid use.

Similarly, the lack of structured drug treatment provisions in the community in many LMIC (e.g., some of the Middle-eastern countries) makes embedding THN potentially more difficult. There are over 30 countries that have the death penalty for drug-related offenses [66, 67], most of which are likely LMIC. As such, fears of litigation may be much higher in these areas. Thus, the realities of working with harm reduction methods when the penalties surrounding drug use are so severe may be completely different than the developed world. Hence, the barriers and the possible solutions and remediation strategies that are delineated in this paper are probably applicable to LMIC but more difficult to overcome considering the severe penalties, the high cost, the legislative differences and the waivers required to successfully bring in THN strategies to LMIC.

## Study limitations

There were some limitations found in the studies included in this systematic review, such as small sample sizes, convenience sampling indicating possible recruitment or selection bias, missing data and attrition issues in prospective cohort studies. Furthermore, many of the studies relied entirely on self-reported surveys or questionnaires, which could have introduced a recall bias.

## Conclusions

Efforts are being made for the expansion and widespread implementation of THN programs in the developed world, while LMIC with high opioid overdose rates are lagging behind. Much more studies are needed with larger sample sizes to provide knowledge about THN status in LMIC, and the challenges faced in its availability, accessibility and utilization. The main barriers to THN strategies that could be applicable to LMIC with regard to the healthcare system were the lack of training of healthcare providers, time constraints, cost, statutory/policy restrictions, and some misperceptions around prescribing THN. On the other hand, stigma, fear of litigation, and other negative beliefs/attitudes were some of the barriers identified from opioid users. Further focus should be directed towards reducing prescribing and regulatory barriers, and providing overdose education and THN training to alleviate these concerns.

## Data Availability

Available, not published.
